# *In vitro* chloroquine resistance for *Plasmodium vivax* isolates from the Western Brazilian Amazon

**DOI:** 10.1186/1475-2875-12-226

**Published:** 2013-07-03

**Authors:** Yonne F Chehuan, Monica RF Costa, Jacqueline S Costa, Maria GC Alecrim, Fátima Nogueira, Henrique Silveira, Larissa W Brasil, Gisely C Melo, Wuelton M Monteiro, Marcus VG Lacerda

**Affiliations:** 1 Fundação de Medicina Tropical Dr. Heitor Vieira Dourado, Av. Pedro Teixeira, 25, Dom Pedro, Manaus, AM, 69040-000, Brazil; 2 Universidade do Estado do Amazonas, Av. Pedro Teixeira, 25, Dom Pedro, Manaus, AM, 69040-000, Brazil; 3 Centro de Malária e Outras Doenças Tropicais, UEI Malária, Instituto de Higiene e Medicina Tropical, Universidade Nova de Lisboa, Rua da Junqueira, 100, Lisbon, 1349-008, Portugal; 4 Universidade Nilton Lins, Av. Prof. Nilton Lins, 3259, Parque das Laranjeiras, Manaus, AM, 69058-030, Brazil

**Keywords:** Malaria, *Plasmodium Vivax*, Chloroquine, Mefloquine, Resistance, Treatment

## Abstract

**Background:**

Chloroquine (CQ) and primaquine (PQ) are still the drugs of choice to treat *Plasmodium vivax* malaria in many endemic areas, Brazil included. There is *in vivo* evidence for the *P. vivax* resistance to CQ in the Brazilian Amazon, where the increase in the proportion of *P. vivax* malaria parallels the increase of unusual clinical complications related to this species. In this study, *in vitro* CQ and mefloquine (MQ)-susceptibility of *P. vivax* isolates from the Western Brazilian Amazon was tested using the double-site enzyme-linked lactate dehydrogenase immunodetection (DELI) assay.

**Methods:**

A total of 112 *P. vivax* isolates were tested *in vitro* for CQ-susceptibility and out of these 47 were also tested for MQ-susceptibility. The DELI assay was used to detect *P. vivax* growth at 48-hour short-term culture in isolates with ring stages ranging from 50 to %. Each isolate was tested in triplicate and geometric means of IC50’s was obtained. Nineteen isolates were genetically characterized for *pvdhfr, pvmrp1, pvmdr1* and *pvdhps* candidate genes likely related to CQ resistance (10 with IC50<40 nM and 9 with IC50 >100 nM).

**Results:**

Twelve out of 112 isolates were considered resistant to CQ, resulting in 10.7% (IC95% 5.0-16.4), while 3 out of 47 (6.4%; IC95% 0.0-12.8) were resistant to MQ. A discrete correlation was observed between IC50’s of CQ and MQ (Spearman=0.294; p=0.045). For *pvdhps* gene, a non-synonymous mutation was found at codon 382 (S→C) in 5/8 CQ-sensitive samples and 1/9 CQ-resistant samples (p=0.027). The other molecular markers were not associated to CQ-susceptibility.

**Conclusions:**

*In vitro* CQ-resistance estimated in this study, estimated by the DELI test, was very similar to that observed in clinical trials, suggesting that *in vitro* procedures developed by capable local laboratories are useful in the surveillance of CQ-resistance in the Amazon; concurrent Amazon *P. vivax* strains with both CQ and MQ resistance may be common; and a non-synonymous mutation at *pvdhps* codon 382 (S→C) was associated to *in vitro* susceptibility to CQ, needing further studies to be confirmed.

## Background

*Plasmodium vivax* remains more widely distributed than *Plasmodium falciparum* and is a potential cause of morbidity and mortality amongst the 2.85 billion people living at risk of infection, the majority of whom are in the tropical belt of Central and Southeastern Asia and Latin America [1]. In Brazil, *P. vivax* is responsible for more than 80% of the cases in the last years [2]. In this country, the increase in the proportion of *P. vivax* malaria parallels the increase in the frequency of unusual clinical complications in *P. vivax-*infected patients [3]. Chloroquine (CQ) and primaquine (PQ) are still the drugs of choice to treat vivax malaria in many endemic areas, Brazil included. The simultaneous occurrence of severe vivax disease and CQ-resistance in some countries has raised the question of a possible association between severity and resistance [4]. CQ-resistance actually has been reported in Brazil almost at the same time as clinical severity [5].

There is *in vivo* evidence for the *P. vivax* CQ-resistance in the Brazilian Amazon. In this region, the resistance to CQ was firstly reported in Manaus in 1999 [5]. More recent data from studies conducted in the RAVREDA multicenter study (*Amazonian Network for Surveillance for Resistance to Antimalarial Drugs*) with a proper follow-up of patients exclusively using CQ and in whom drug plasma concentration was evaluated, seem to confirm such an observation [5]. However, since CQ plus PQ, the drug association used for the radical cure of *P. vivax* infection, has a synergistic action [6], further studies are needed to evaluate the contribution of CQ by itself for the effectiveness of the drug association, before any definitive conclusion can be proposed.

*In vitro* surveillance of anti-malarials resistance is largely used in *P. falciparum* with public health purposes, however *P. vivax* can grow only to a certain extent under *in vitro* conditions, with a very low reinvasion rate, what poses technical limitations to the routine use of this approach. The first *in vitro P. vivax* short-term culture was performed in 1974 [7], but more recent publications have presented reproducible results based on the WHO microtest using quantification of schizont maturation [8]. The method is cheap, however it is time-consuming since slides from the drug-free well have to be made every 6 hours trying to achieve the moment when there is schizonts maturation of 40%. Even demanding monoclonal antibodies, what makes it more expensive, the double-site *Plasmodium* lactate dehydrogenase (LDH) antigen capture enzyme-linked immunosorbent assay (DELI) [9] quantifies the total pLDH produced by maturing stages, and can be performed in 48 hours after the beginning of the culture, avoiding sequential slides reading. The aim of this study was to estimate the *in vitro* CQ-susceptibility of *P. vivax* isolates from the Western Brazilian Amazon using the DELI assay.

## Methods

### Site of study and patients

The study was performed from December 2007 to July 2008 in the Fundação de Medicina Tropical Dr. Heitor Vieira Dourado (FMT-HVD), which reports 30% of all the malaria cases in Manaus (03°06′S, 60°01′W). Patients living in the urban or peri-urban areas of this city with uncomplicated *P. vivax* malaria confirmed by a thick blood smear (TBS) were randomly selected in the outpatient clinics, from which epidemiological and clinical history was fully obtained. Parasite densities with differential counting of rings, trophozoites and schizonts were estimated by experienced microscopists, by counting the number of parasites in 200 leukocytes in high magnification fields, and the number of leukocytes/mm^3^. The study included patients of both sexes, aged 12–60 years, presenting blood parasite density from 250 to 100,000 parasites/mm^3^ and axillary temperature ≥ 37.5°C or history of fever in the last 48 hours. The major exclusion criterion was the use of anti-malarials in the previous 60 days. Due to the selective action of CQ upon young *P. vivax* trophozoites (rings) [10], only samples with ring stages ranging from 50-70% at the initial counting were analysed in this study.

### *In vitro* determination of susceptibility to drugs

Blood samples were collected in on Vacutainer (Becton Dickinson®, Oxnard, CA) EDTA tubes prior to patient treatment. CQ sulfate and MQ hydrochloride were obtained from Sigma-Aldrich®. Isolates from the same outpatient clinics were randomly selected (47 were tested for CQ and MQ susceptibility and additional 65 samples were tested only for CQ). The blood samples were washed twice with a solution of RPMI-1640 Medium (Gibco®) and then one time with culture complete medium. The blood samples were then resuspended in the complete culture medium to obtain a 1.5% haematocrit. Finally, 200 μL of this suspension were distributed to each well in the anti-malarial pre-dosed plates. The plates were systematically incubated for 48h at 37°C (5% CO_2_, 5% O_2_ and 90% N_2_) and were immediately frozen at −20°C until the DELI test was performed with many samples at the same time. Only cultures with viable schizonts at 48 hours were considered valid. For the DELI test, plates were frozen and thawed three times to haemolyse the culture and release pLDH [9]. Each isolate was tested in triplicate and geometric means of IC50’s was obtained. IC50 threshold criterion for resistance to CQ or MQ was similar to that described elsewhere [9]. Dd2 and 3D7 *P. falciparum* clones were used as controls.

### Molecular characterization

Nineteen isolates were genetically characterized for a few candidate genes linked to CQ resistance (10 with IC50<40 nM and 9 with IC50 >100 nM). Whole DNA extraction was carried out using a QIAamp® DNA Mini Kit (QIAGEN®, Germany) according to the manufacturer’s protocol. PCR primers and different reaction conditions used to amplify *P. vivax* dihydrofolate reductase (*pvdhfr*)*,* multidrug resistance-associated protein 1 (*pvmrp1*)*,* multidrug resistance 1 *(pvmdr-1)* locus, and dihydropteroate synthase (*pvdhps*) gene sequences were made as previously described [11-16] (Additional file 1). Briefly, after initial denaturing at 94°C for 2 minutes, the samples were submitted to 35 cycles (94°C for 1 minute more, 58°C for 30 seconds, and 72°C for 1 minute), with a final extension at 72°C for 10 minutes. For each fragment, PCR products were visualized by 1% agarose gel electrophoresis stained with ethidium bromide to confirm single band. DNA concentration was measured by NanoDrop® 2000 (Thermo Scientific®). Sequencing reactions were carried out using an ABI 3130xl genetic analyzer (Applied Biosystems®, USA) as specified by manufacturer’s protocol.

### Data analysis

IC50 was calculated in the software Analysis of Malaria In Vitro Drug Sensitivity Data (HN-NonLin V 1.05 Beta®). Correlation between CQ and MQ IC50 was calculated through Pearson test. Fisher’s test was employed to evaluate the association between point mutations and CQ-resistance. Analysis was performed using SPSS software for Windows (version 16; SPSS, Inc., Chicago, IL®). P<0.05 was considered significant.

### Ethical procedures

Collection of human samples and all study procedures were approved by the Ethical Review Committee of Fundação de Medicina Tropical Dr. Heitor Vieira Dourado (protocol number 1850/2007). Written informed consent was obtained from all the participant patients. All malaria cases were treated according to the Brazilian Ministry of Health’s National guidelines, with CQ and PQ.

## Results

**Figure 1:**
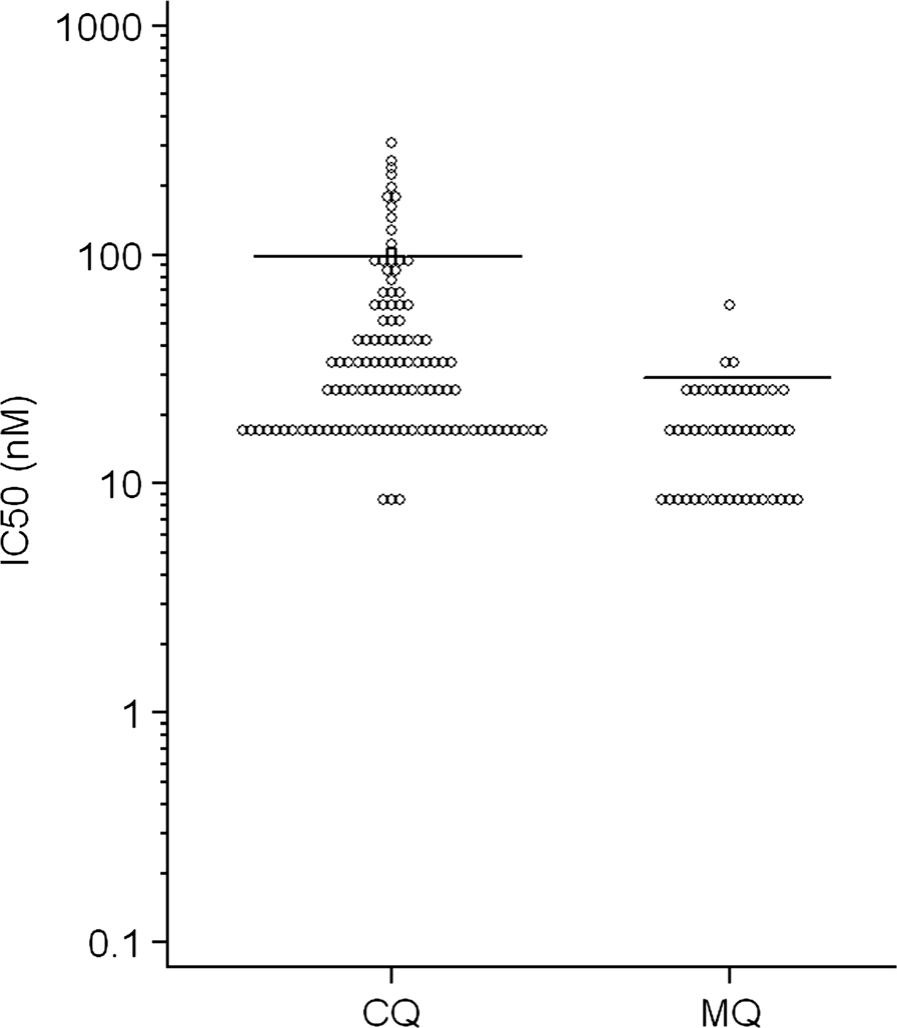
**Distribution of IC50’s (nM) among 112 *Plasmodium vivax *isolates from Manaus cultured in the presence of various concentrations of chloroquine (CQ) or mefloquine (MQ).** The horizontal bars represent the likely thresholds between sensitivity and resistance.

**Figure 2:**
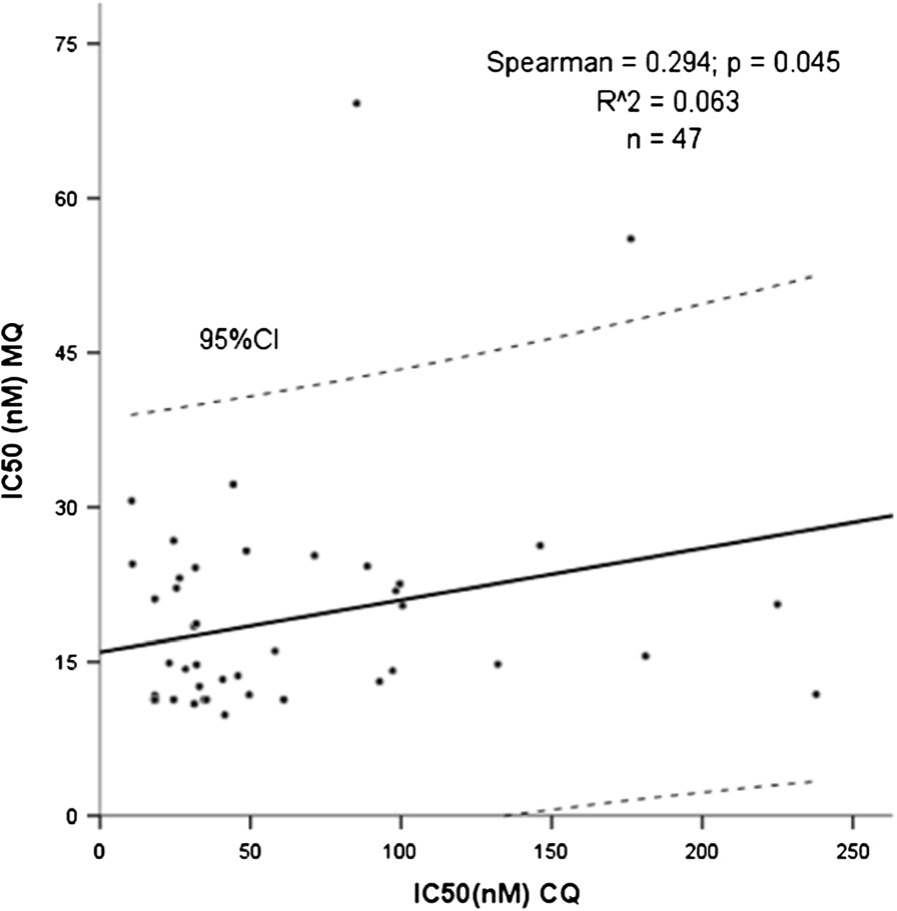
**Spearman correlation between IC50’s of chloroquine and mefloquine for *Plasmodium vivax *isolates.**

Twelve out of 112 isolates were considered resistant to CQ, resulting in 10.7% (CI95% 5.0-16.4) of CQ-resistance, while three out of 47 (6.4%) (CI95% 0.0-12.8) were resistant to MQ (Figure 1). A discrete correlation was observed between IC50’s of CQ and MQ (Spearman=0.294; p=0.045) (Figure 2).

As seen in the Table 1, for *pvdhps* gene, a non-synonymous mutation at codon 382 (S→C) was found in 5/8 samples *in vitro* sensitive and 1/9 samples *in vitro* resistant to CQ (p=0.027). The other molecular markers were not associated to CQ susceptibility.

**Table 1:** **Frequency of polymorphisms in the *pvdhfr, pvmrp1, pvmdr1 and pvdhps *genes for sensitive and resistant *in vitro *samples**

**Gene/Polymorphisms**	**Sensitive samples**	**Resistant samples**	**p-value**
***Pvdhfr***			
58	7/10 (70%)	8/9 (88.9%)	0.313
117	8/10 (80%)	8/9 (88.9%)	0.596
173	1/10 (10%)	0/9 (0%)	0.330
***pvmrp1***			
1282	9/9 (100%)	7/7 (100%)	NS
1393	9/9 (100%)	7/7 (100%)	NS
1419	4/9 (44.4%)	2/7 (28.6%)	0.515
1478	10/10 (100%)	9/9 (100%)	NS
1586	2/10 (20%)	2/9 (22.2%)	0.906
***pvmdr1***			
908	1/10 (10%)	1/9 (11.1%)	0.937
958	10/10 (100%)	8/9 (8.9%)	0.279
***Pvdhps***			
205	10/10 (100%)	9/9 (100%)	NS
382	5/8 (62.5%)	1/9 (11.1%)	0.027
383	0/8 (0%)	1/9 (11.1%)	0.331

*NS* not significant.

## Discussion

*Plasmodium vivax in vitro* CQ-resistance levels in Latin America are unknown. In 2007, the proper 28 days follow-up of 109 patients with *P. vivax* prescribed only with CQ lead to the unexpected confirmation of 10.1% of resistance after plasmatic drug dosage [17], a rate very similar to that obtained from this study, suggesting that *in vitro* procedures developed by capable local laboratories are useful to the surveillance of CQ-resistance in the Amazon.

An interesting finding of this study was the concurrent resistance to both CQ and MQ within *P. vivax* strains from Amazon. In this region, a CQ-resistance rate of around 10% requires the evaluation of alternative drugs such MQ. Although MQ was thought to be a useful alternative treatment for *P. vivax* malaria in [18] and indirect evidence suggested that this drug may be efficacious against CQ-resistant *P. vivax* in Indonesian New Guinea [19]*,* there is no previous information from Latin America supporting these data. In 1999, Alecrim *et al.*[5] reported a case of a patient with *P. vivax* malaria who showed resistance to chloroquine and mefloquine in the Brazilian Amazon region. CQ and MQ-resistance overlap have also had commonly reported from Thailand [20]. Nomura [21] pointed to different mechanisms of resistance between the two species, however some data including results presented here show that caution is required.

Among the isolates with IC50 defined, 19 have been genetically characterized (10 with IC50<40 nM and nine with IC50 >100 nM). For *pvdhps* gene, a non-synonymous mutation was found at codon 382 (S→C) in 5/8 samples *in vitro* sensitive and 1/9 samples *in vitro* resistant (p=0.027). It is known that the spread of sulphadoxine-pyrimethamine in *P. falciparum* exerted selective pressure for *pvdhps* mutations in *P. vivax.* Molecular studies on a population basis for *P. falciparum* have demonstrated that patients who were infected with parasites that carried this mutation may not respond to treatment [14], but when the parasite carries highly mutant versions of *pvdhps* and *pvdhfr* genes, clinical effectiveness is compromised [22]. Another study showed that resistance-conferring mutations were not found in *pvdhps*[23]. The other genetic markers were not associated with drug response. Suwanarusk *et al.*[24] studying the same gene, found that CQ IC50 was significantly higher in *P. vivax* isolates carrying the Y976F mutation than in isolates with the wild-type allele. Ranjitkar *et al.*[25] found that the low resistance to CQ was due to the low prevalence of mutation Y976F in *pvmdr1* (5%). However, most studies did not find any association between mutation in the *pvmdr-1* gene and CQ-resistance corroborating these results [16,21,26,27].

Major limitations of this study were: (1) the small number of samples; (2) the lack a standard cut-off IC50 for the characterization of resistance in Latin American samples; (3) DELI test is a less time-consuming test due to the detection of pLDH in 48-hour culture plates, however it was never validated as compared to the microscopy test; (4) *in vitro* resistance was not individually compared to *in vivo* resistance. On the other hand, standard techniques are not easily reproducible among the different scenarios in endemic areas, due to personnel, reagents laboratory conditions. Data from the same laboratories over time is more reliable information and may be used as a baseline for future further studies using the same technique. This paper presents preliminary data on the *in vitro P. vivax* resistance in the Western Amazon. More information is needed regarding the usefulness of this approach in detecting *in vivo* resistance and the association of more severe cases.

## Conclusion

In summary, this study has shown that: 1) *in vitro* CQ-resistance estimated in this study was very similar to that obtained from clinical trials in the same area [17], suggesting that *in vitro* procedures developed by capable local laboratories are useful in the surveillance of the CQ-resistance in the Amazon; 2) *P. vivax* strains simultaneous resistance to both CQ and MQ within in the Amazon needs to be clarified; and 3) a non-synonymous mutation at *pvdhps* gene codon 382 (S→C) was associated to *in vitro* susceptibility to CQ.

The high levels of anti-malarial drug resistance in this region call for reinforced surveillance of drug efficacy. *Plasmodium vivax* CQ-resistance may be rising in the Brazilian Amazon and probably this fact is contributing to spread vivax malaria and clinical severity of this disease. However, the public health urgency to detect and measure the progression of CQ-resistant vivax malaria has been neglected. To maintain an efficient malaria control programme, drug resistance surveillance assays must be conducted on a regular basis to assess anti-malarial efficacy and to ensure that the information is available to policy makers.

## Competing interests

The authors declare that they have no competing interests.

## Authors’ contributions

YFCM, MRFC, JSC, FN, LWB and GCM participated in laboratory procedures. YFCM, MRFC, MGCA, HS, WMM and MVGL participated in overall study conception and design, data collection, analysis, interpretation and manuscript preparation. All authors read and approved the final manuscript.

## Supplementary Material

Additional file 1 **Oligonucleotide primers used for PCR amplification and DNA sequencing of *Plasmodium vivax *genes.** Click here for file
