# Supplementary material for: In vitro chloroquine resistance for Plasmodium vivax isolates from the Western Brazilian Amazon
Source: Malar J. 2013 Jul 3;12:226. doi: 10.1186/1475-2875-12-226 (PMC3704965; doi:10.1186/1475-2875-12-226)
Supplement: Additional file 1 — Oligonucleotide primers used for PCR amplification and DNA sequencing of Plasmodium vivax genes. [file 1475-2875-12-226-S1.docx]

Additional file 1. Oligonucleotide primers used for PCR amplification and DNA sequencing of *Plasmodium vivax* genes.

| **Gene** | **Chromosome** | **Primer pair** | **Sequence 5’🡪3’** | **Base pairs** | **Mutations sought** | **References** |
| --- | --- | --- | --- | --- | --- | --- |
| ***pvdhfr*** | 5 | PV1F | CAGTGAAGGGACAAAGAATGAACC | 560 bps | S58R/S117N/I173L | Barnadas *et al*. [1]  de Pecoulas *et al*. [2]  Hawkins *et al.* [3] |
|  |  | PV1R | ACTCGGGGAAGAAGACGTCAC |  |  |  |
| ***pvmrp1*** | 2 | PV5F | CATATCGGGAAAAAGCGTAATTAACG | 523 bps | L1282I /Y1393D /G1419A /V1478I /H1586Y | Raj *et al*. [4] |
|  |  | PV5R | CTTCGATTGGTCTATGGCTGGTG |  |  |  |
|  |  | PV6F | TCGAGAACGTATTCGTCAGTTATAAG | 497 bps |  |  |
|  |  | PV6R | GTTGCTCGAAAGGTTAGCCTTTC |  |  |  |
| ***pvmdr1*** | *10* | PV7F | GCCATGTTCATTTCTGAGACGCTG | 337 bps | M908L/L958M | Barnadas *et al*. [5]  Orjuela-Sanchez *et al*. [6] |
|  |  | PV7R | TCGCTCTGATGGCAAACACTC |  |  |  |
| ***pvdhps*** | *14* | PV8F | TTTTAAAGTACATTGAGCAAATCGTG | 200 bps | S382C/A383G | Barnadas *et al.* [1]  Hawkins *et al*. [3] |
|  |  | PV8R | CTGATCACTTGTGTGGTTTATGTG |  |  |  |
|  |  | PV9F | GCGGTTTATTTGTCGATCCTGTG | 244 bps |  |  |
|  |  | PV9R | TTTTTCCTGGCATCACTTGCTG |  |  |  |

**References**

1. Barnadas C, Musset L, Legrand E, Tichit M, Briolant S, Fusai T, Rogier C, Bouchier C, Picot S, Menard D: **High prevalence and fixation of *Plasmodium vivax* dhfr/dhps mutations related to sulfadoxine/pyrimethamine resistance in French Guiana**. *Am J Trop Med Hyg* 2009, **81**:19–22.

2. de Pecoulas PE, Tahar R, Ouatas T, Mazabraud A, Basco LK: **Sequence variations in the *Plasmodium vivax* dihydrofolate reductase-thymidylate synthase gene and their relationship with pyrimethamine resistance**. *Mol Biochem Parasitol* 1998, **92**:265–273.

3. Hawkins VN, Joshi H, Rungsihirunrat K, Na-Bangchang K, Sibley CH: **Antifolates can have a role in the treatment of *Plasmodium vivax***. *Trends Parasitol* 2007, **23**:213–222.

4. Raj DK, Mu J, Jiang H, Kabat J, Singh S, Sullivan M, Fay MP, McCutchan TF, Su XZ: **Disruption of a *Plasmodium falciparum* multidrug resistance-associated protein (PfMRP) alters its fitness and transport of antimalarial drugs and glutathione**. *J Biol Chem* 2009, **284**:7687–7696.

5. Barnadas C, Ratsimbasoa A, Tichit M, Bouchier C, Jahevitra M, Picot S, Menard D: ***Plasmodium vivax* resistance to chloroquine in Madagascar: clinical efficacy and polymorphisms in pvmdr1 and pvcrt-o genes**. *Antimicrob Agents Chemother* 2008, **52**:4233–4240.

6. Orjuela-Sanchez P, de Santana Filho FS, Machado-Lima A, Chehuan YF, Costa MR, Alecrim MG, del Portillo HA: **Analysis of single-nucleotide polymorphisms in the crt-o and mdr1 genes of *Plasmodium vivax* among chloroquine-resistant isolates from the Brazilian Amazon region**. *Antimicrob Agents Chemother* 2009, **53**:3561–356.
